# Mutation Accumulation May Be a Minor Force in Shaping Life History Traits

**DOI:** 10.1371/journal.pone.0034146

**Published:** 2012-04-06

**Authors:** Maciej Jan Dańko, Jan Kozłowski, James Walton Vaupel, Annette Baudisch

**Affiliations:** 1 Max Planck Institute for Demographic Research, Rostock, Germany; 2 Institute of Environmental Sciences, Jagiellonian University, Kraków, Poland; Centers for Disease Control and Prevention, United States of America

## Abstract

Is senescence the adaptive result of tradeoffs between younger and older ages or the nonadaptive burden of deleterious mutations that act at older ages? To shed new light on this unresolved question we combine adaptive and nonadaptive processes in a single model. Our model uses Penna's bit-strings to capture different age-specific mutational patterns. Each pattern represents a genotype and for each genotype we find the life history strategy that maximizes fitness. Genotypes compete with each other and are subject to selection and to new mutations over generations until equilibrium in gene-frequencies is reached. The mutation-selection equilibrium provides information about mutational load and the differential effects of mutations on a life history trait - the optimal age at maturity. We find that mutations accumulate only at ages with negligible impact on fitness and that mutation accumulation has very little effect on the optimal age at maturity. These results suggest that life histories are largely determined by adaptive processes. The non-adaptive process of mutation accumulation seems to be unimportant at evolutionarily relevant ages.

## Introduction

The evolution of senescence is explained by two main approaches: a non-adaptive theory (mutation accumulation [1]) and adaptive theories (antagonistic pleiotropy [2], disposable soma [3]). Generally, the first approach assumes that senescence is an evolutionary burden due to late-acting deleterious mutations that accumulate in the germline, whereas the second assumes that senescence is a negative byproduct of an adaptive process constrained by tradeoffs of early benefits against less important later costs. Both approaches rest on the observation that the force of selection declines with age. As quantified by Hamilton [4], the selection pressure on changes in mortality at some particular age in a non-growing population is proportional to remaining reproduction at that age. Remaining reproduction is captured by the sum of age-specific reproductive contributions, weighted by the probability of being alive at that age. The proportion of reproduction remaining to an organism at some age inevitably dwindles over adult ages from 100% at reproductive maturity to 0% at the age of last reproduction. Assuming that mutation pressure is the same across ages, mutations that take their affect at late ages accumulate at higher frequencies because of the declining force of selection.

Which of the two complementary processes – adaptive or non-adaptive – is more important in explaining senescence? This is still a major, unresolved question that has spurred important empirical and theoretical work [5] [12].

Reliable empirical evidence for adaptive explanations seems stronger than for non-adaptive ones: tradeoff processes have received wide experimental support, in the lab [5] and in the wild [13] [16], as reviewed by Partridge and Barton [7] and more recently by Flatt and Promislow [17]. Evidence supporting mutation accumulation has been found [18], [19], [9], [20], but the conclusiveness of this evidence has been questioned because it is difficult to empirically distinguish between mutation accumulation and antagonistic pleiotropy [21], [22], [9]. Recent work suggests that current methods to do so are not decisive [12].

Several theoretical models have been developed to explain how mutation accumulation shapes mortality patterns (e.g., [23], [24] and [10]). We are, however, aware of only one theoretical model, developed by Charlesworth [21] that incorporates mutation accumulation within the framework of life history tradeoffs. Though he concludes that “in principle, the accumulation of age-specific mutations can cause a senescent decline in life-history traits”, the question of whether senescence is mainly a by-product of evolution optimizing life history traits within given constraints or mainly results from mutation accumulation, remains unanswered. To answer this question, we combine mutation accumulation and life history optimization in one model within a framework that is different from (yet, as we will show below, complementary and consistent with) the quantitative genetics approach applied by Charlesworth. In our framework, the genome of a species is represented by a “bit-string,” an approach popularized in biology as the Penna Model [25] (see [26] for review).

## Methods

We assume a non-growing population with non-overlapping generations of one-sex haploids facing constant background mortality. As in the Penna Model, individuals are represented by their genome. The genome is given by a string of zeros and ones. Zero stands for a non-mutated gene and one stands for its mutated variant. In our model, genes code for the level of mortality at consecutive ages. Genes may also have an effect on reproduction, as will be discussed later.

Following the basic corollary of mutation accumulation theory (genes responsible for senescence have age-specific effects on fertility and mortality), in our model genes are expressed at the beginning of their respective age-intervals. Their action persists until the end of life. The age of expression of the gene is assumed to be equivalent to the age when the gene's effect becomes apparent in changing mortality. A genotype with no mutations at any loci (i.e., corresponding to a vector of zeros only) experiences constant mortality, equal to the constant background death rate 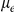. We assume that mutated gene increases mortality additively by a constant 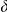 from that age onwards (an additive, cumulative effect as in e.g., [23]). When there is no mutation in an age-specific gene, the death rate stays at the same level as in the previous age interval. Thus, mortality for genotype *g* at age *x* is given by *μ_x_(g) = π(x,g) δ+μ_e_*, where *π(x,g)* captures the number of expressed mutations at age *x*.

The maximum age 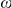 in the population is set to the age when remaining reproduction falls below 0.0001 for the non-mutated genotype, since ages beyond that point do not significantly alter fitness for any genotype (see e.g., [27] and [28]). As in the Penna Model, we divide ages into equal intervals, usually 10 in our model. Assuming 10 genes proved to be the best compromise between model precision and computing demands.

We assume infinite population size. Hence the expected frequency of each genotype can be determined exactly. As an illustration, imagine the case of only two loci with frequencies of deleterious alleles *p_1_* and *p_2_* in a given generation. Four genotypes are possible: genotype **[00]** with no gene mutated, genotype **[10]** with the first locus mutated, genotype **[01]** with the second locus mutated, and genotype **[11]** with both loci mutated. Table 1 shows how the distributions of these genotypes can be converted into frequencies *p_1_* and *p_2_* of mutated genes. Our simulation begins with an initial distribution of genotypes. The choice of a particular initial distribution did not affect the results of the model (see Results). From generation to generation, frequencies of genes change, depending on the fitness of each genotype. We assume that all individuals are born at the same initial size, independent of genotype. Following standard life history approaches (e.g., [27] and [29] [33]) size, *W(t)*, at age *t* changes according to
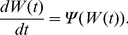
(1)where *Ψ(W)* denotes production rate, i.e., the rate at which surplus energy can be allocated to growth and/or reproduction. Production rate is assumed to be an increasing and concave function of size,
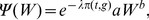
(2)with constant, non-negative parameters *a* and *b<1*, assuring diminishing returns from increase of body mass. Parameter *a* is set to 0.2. It scales size and time units in the model, which are not the focus of our analysis. Parameter *b* is set to 0.75, as is typically done in similar models (e.g., [34] [–] [36]).

**Table 1:** Converting a distribution of genotypes into a distribution of mutations: Example for 2-locus case.

Genotype		Frequency of genotype	Contribution of each genotype to frequency of mutated locus	
L1	L2		L1	L2
0	0	Q [00]	0	0
0	1	Q [01]	0	Q[01]
1	0	Q [10]	Q [10]	0
1	1	Q [11]	Q [11]	Q [11]
Frequency of mutations at L1 and L2:			*p*_1_ = Σ(…)	*p*_2_ = Σ(…)

Note that *p*_1_ and *p*_2_ are frequencies of mutations at loci L1 andL2. 2-locus case is shown for simplicity, but at least 10 locus cases are considered in the paper.

Unlike standard approaches, we incorporate an exponential factor that captures the effect of an individual's genotype on energy production. We assume that mutations influence energy production of genotype *g* via their number *π(t,g)* expressed at age *t* and via the strength of their effect as captured by the constant, non-negative parameter *λ*. Thus, genotypes with more mutated and expressed at age *t* genes are less efficient in producing its own or offspring tissues at the age *t*.

Energy calculated according to (2) can be allocated to growth or to reproduction. Previous models of this kind have shown that the optimal resource allocation strategy in such a setting is to invest all energy into growth until reproductive maturity and thereafter to switch all allocation to reproduction [37] (see [33] for review). The same holds true for our modified model including an exponential factor. Hence the optimal life history strategy is characterized by a single life history trait, the optimal age at maturity, denoted by 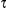.

Size at maturity can be calculated solving (2):
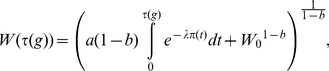
(3)where 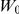 is the size of a newborn individual, which is set to one. After maturity it is optimal to invest all energy into reproduction: the reproductive rate per one time unit at age *t* is given by
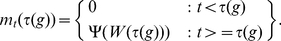
(4)Reproductive output is equivalent to the amount of energy invested in reproduction. Note that energy is measured in units equivalent to offspring size because it is normalized to one.

The benefit of a later age at maturity 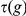 is a larger adult size and so larger amount of energy that can be devoted to reproduction and the cost is a lower chance of surviving until maturity. Note that for *λ* = 0, mutations do not affect production rate and thus the rate of reproduction. In this case, reproduction is constant over adult ages. Otherwise, if *λ*>0, then reproduction declines with age as later acting mutations are expressed, reducing the efficiency of reproductive energy production.

As extensively discussed elsewhere, the appropriate measure of fitness for this kind of life history model is reproductive value at birth [38], [39], which for organisms living in a stationary population reduces to the net reproductive rate, here denoted as *R*(*g*) for a genotype *g*,
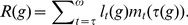
(5)The function 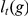 captures the probability of surviving from birth to age *t* for a genotype *g*. It is determined by its cumulative mortality experienced until age *t*,
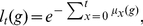
(6)where mortality for genotype *g* at age *x* is given by *μ_x_(g) = π(x,g) δ+μ_e_*.

The algorithm to determine the effect of mutational load on the optimal life history pattern can be described as follows. Given an initial frequency distribution of genotypes, for each specific genotype *g* we calculate the optimal age at maturity *τ(g)* that maximizes fitness *R* (*g*). Gene frequencies *Q*(*g*) are determined by the routine exemplified in Table 1.

**Figure 1:**
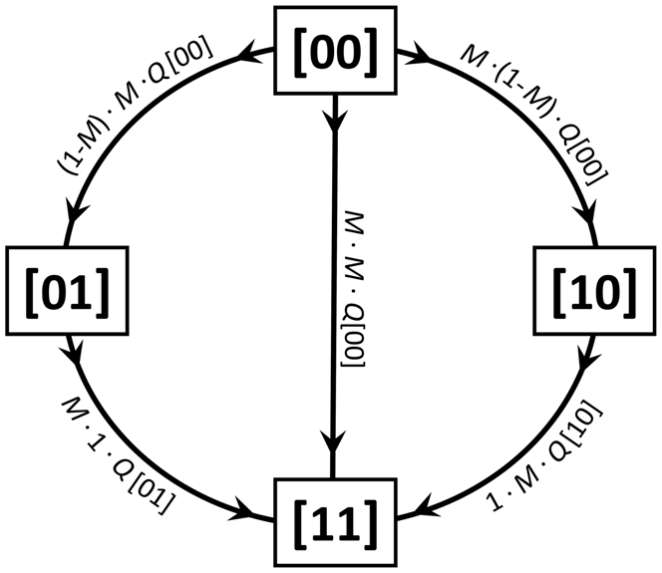
Illustration of changes in frequencies of particular genotypes due to mutations for the exemplary 2-locus case. The mutation rate per locus is constant and equals *M*. Backward mutations are neglected. For example, genotype **[10]** can only mutate into genotype **[11]** (with probability *M*) or remain the same (with probability 1−*M*). The probability of changing the genotype is multiplied by current frequency of the respective genotype to calculate the new frequency. The frequency of each genotype decreases due to mutations in this genotype and increases due to mutations in other genotypes. The change in frequency of genotype **[10]** is equal to the inflow from genotype **[00]** minus the outflow to genotype **[11]**; thus the new frequency ***Q*** *[10] equals ***Q*** **[10]**+*M*·(1−*M*)·***Q*** **[00]**−1·*M*·***Q*** **[10]**. 2-locus case is shown for simplicity, but at least 10 locus cases are considered in the paper.

Two processes alter gene frequencies from generation to generation: selection and mutation. Selection is driven by the genotype-specific fitness *R(g)* and gene frequencies *Q*(*g*). The new distribution of genotypes in the next generation after selection but before mutation, Q*(*g*), can be found by multiplying each frequency *Q(g)* by the expected number of offspring *R*(*g*) and normalizing these results to frequencies:
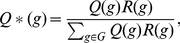
(7)where *G* is the set of all possible genotypes (see also Table 2). Mutations then alter the frequencies of alleles and thus the distribution of genotypes. We assume a constant probability *M* of mutation per locus. Back mutations, being usually orders of magnitude less frequent, are neglected in the model. The transition graph presented in Fig. 1 for the case of two loci exemplifies the procedure to calculate genotype frequencies after mutation.

**Table 2:** Effect of selection on genotypes frequencies.

Genotype		Frequency of genotype	Fitness of genotype	Product	New frequency Q*
L1	L2				
0	0	Q [00]	R [00]	Q [00]R [00]	Q [00]R [00]/*Z*
0	1	Q [01]	R [01]	Q [01]R [01]	Q [01]R [01]/*Z*
1	0	Q [10]	R [10]	Q [10]R [10]	Q [10]R [10]/*Z*
1	1	Q [11]	R [11]	Q [11]R [11]	Q [11]R [11]/*Z*
Normalization factor:				*Z* = Σ(…)	

Example for 2-locus case.

These two routines, selection and mutation, are repeated until equilibrium frequencies of genotypes are reached. The equilibrium condition is fulfilled when the sum of absolute differences between two distributions of genotypes from two consecutive generations is lower than 0.00000001. Once an equilibrium distribution of genotypes is obtained, mean mortality at age *x* in the population can be calculated as
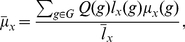
(8)where 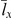 is mean chance of surviving to age x:
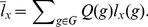
(9)Mutational load measures the decrease of average fitness in a population and can be calculated using the Crow and Kimura [40] equation
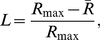
(10)where 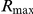 is maximum fitness among genotypes (i.e., for the non-mutated genotype in our model) and 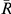 is mean fitness for all genotypes present in the population:
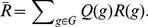
(11)The mean optimal age at maturity 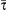 is given by
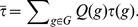
(12)


## Results

We tested our model under different combinations of per locus mutation rates, *M* = 0.0001, 0.001 and 0.01, different effects of mutations on mortality, 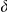 = 0.001, 0.01 and 0.1, different effects of mutations on the reproduction rate, 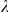 = 0, 0.1 and 1, and different levels of background death rates, 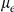 = 0.01, 0.02 and 0.03. As discussed later, we believe that these values of parameters cover the range likely to occur in nature. For each set of parameters we tested three different initial distributions of genotypes: (i) no mutations, (ii) 50% of accumulated mutations in each locus and (iii) 90% of accumulated mutations in each locus. Equilibrium was reached for all possible combinations of parameters (not shown). The initial gene frequencies had no effect on the resulting frequency distribution of genotypes in mutation-selection balance, i.e., the population reaches the same equilibrium independently of initial conditions.

**Figure 2:**
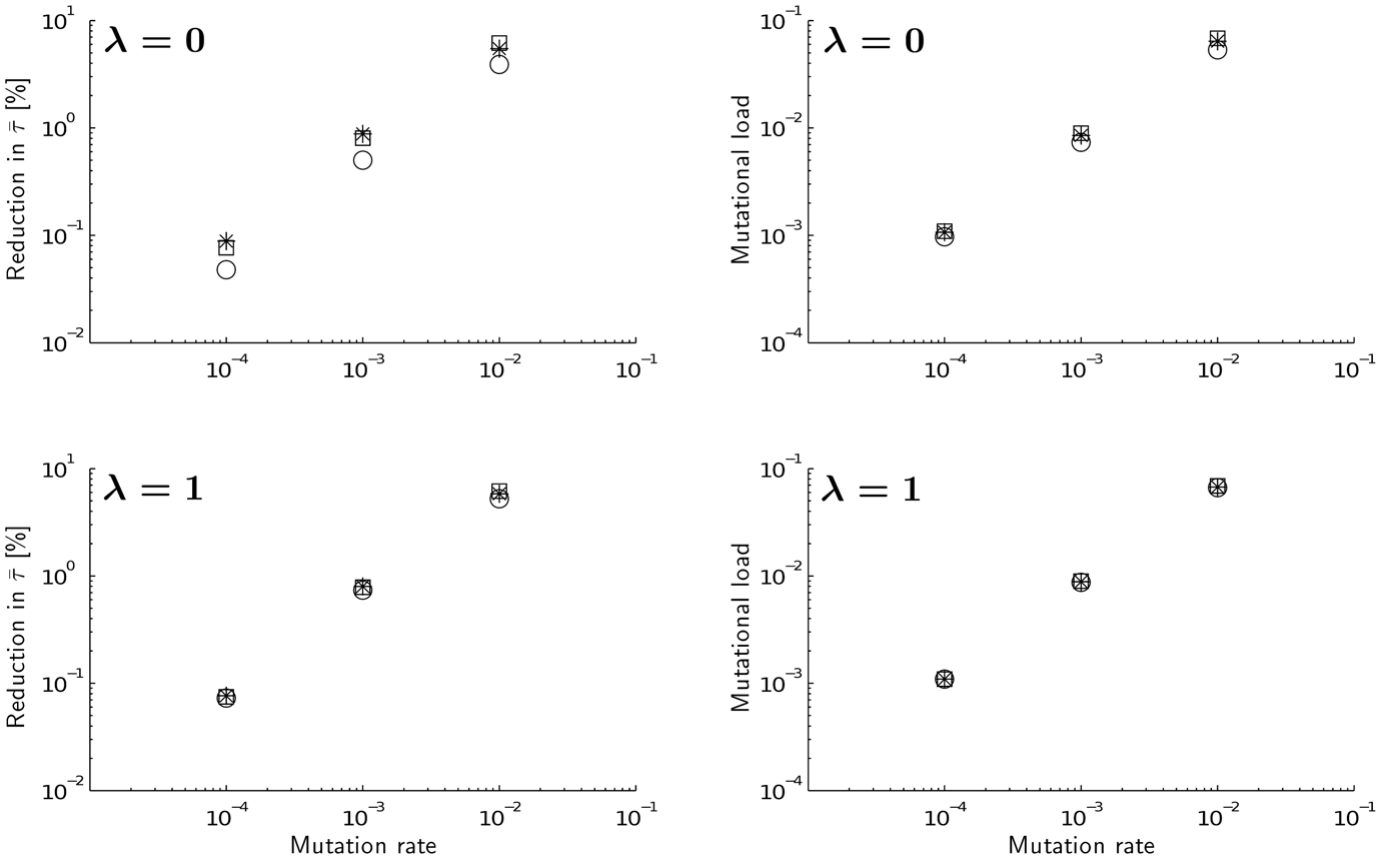
Reduction in mean optimal age at maturity 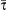
 **due to mutation accumulation (left column) and mutational load (right column) under different levels of the adverse effect of mutations on reproduction** ***λ*** **.** Background mortality *μ_e_* = 0.01 for all graphs. The different levels of adverse effects of mutations on mortality are captured by circles (*δ* = 0.001), stars (*δ* = 0.01) and squares (*δ* = 0.1).

Figure 2 reveals that mutation accumulation does not greatly alter the optimal age at maturity (left-hand column) and does not increase mutational load very much (right-hand column). Compared to the non-mutated genotype, the largest reduction in mean optimal age at maturity, observed for the highest mutation rates, was less than 7%. Moderate and small mutation rates reduced mean maturity by less than 1% and 0.1%, respectively. Very similar impacts were observed for mutational load. Altering the effect (*δ*) of mutations on mortality or the effect (*λ*) of mutations on the reproduction rate did not significantly affect the results.

**Figure 3:**
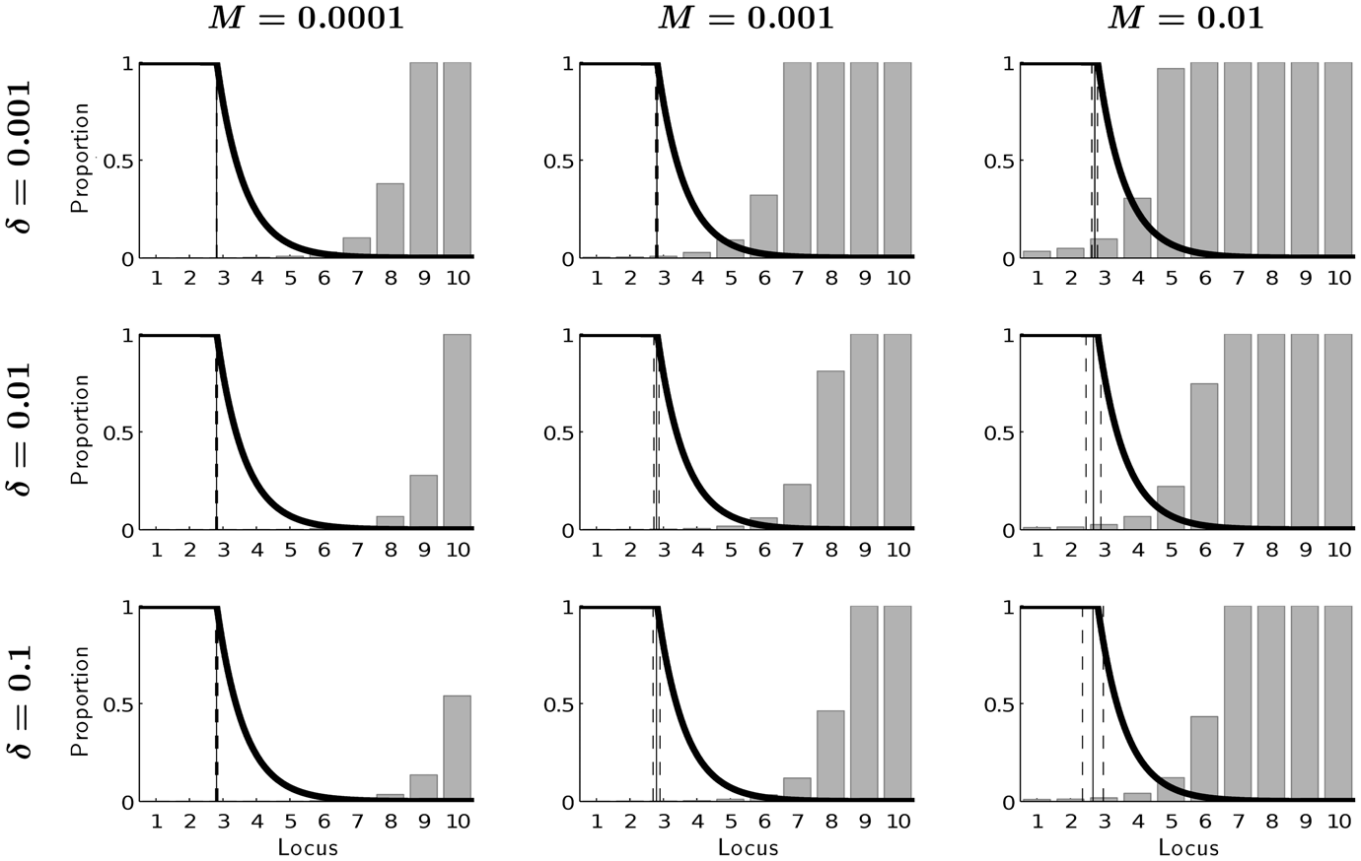
Equlibrium frequencies of mutations at different loci for background mortality *μ_e_* = 0.01 and no effect of mutations on production rate (*λ* = 0) under different mutation rates *M* and different effects of mutations on mortality *δ*. The vertical lines represent mean age at maturity (solid) with standard deviation (dashed). The thick solid line captures the fraction of remaining reproduction, which is proportional to Hamilton's force of selection.

**Figure 4:**
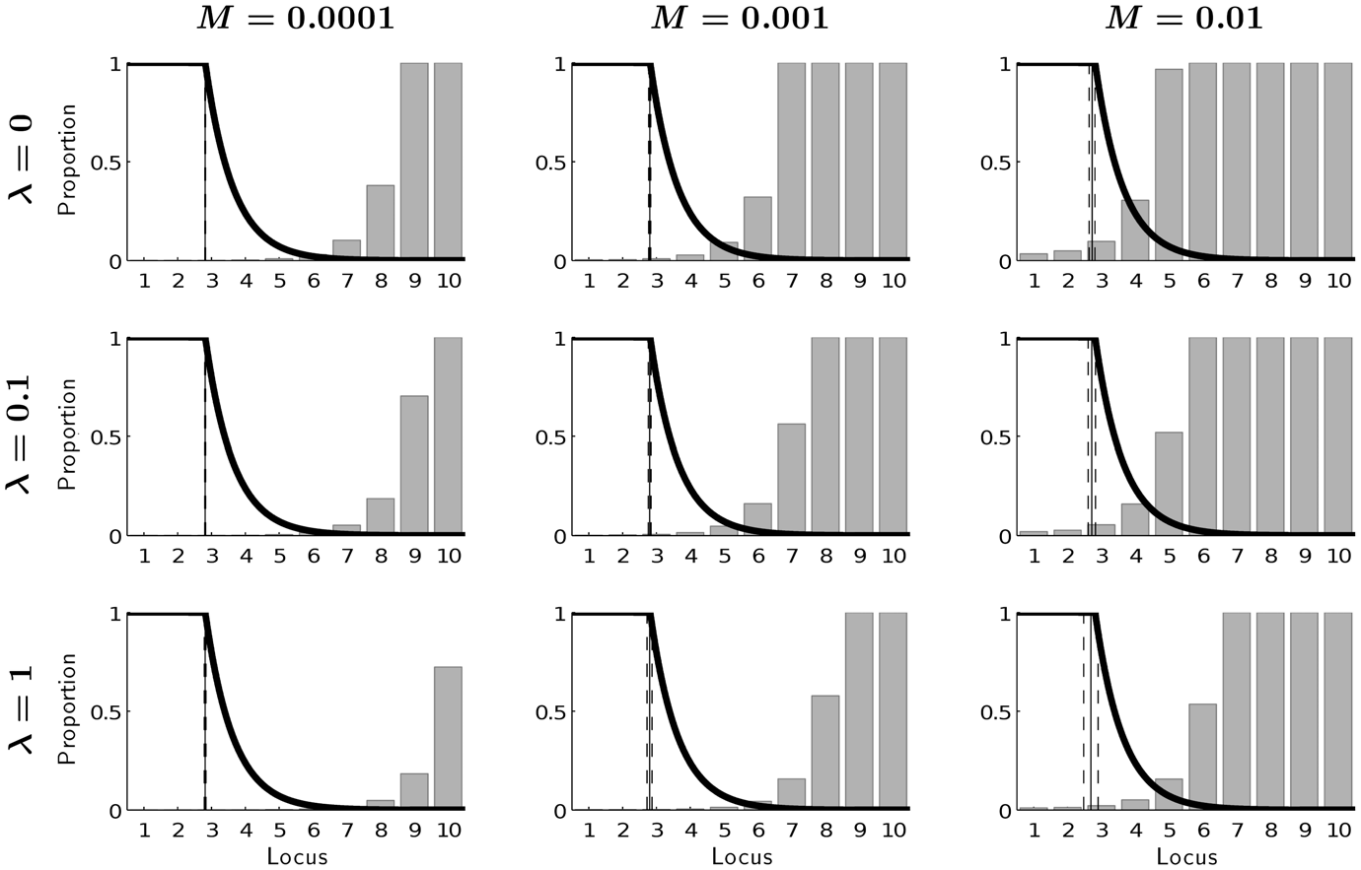
Equilibrium frequencies of mutations in different loci for background mortality *μ_e_* = 0.01 and adverse effect of mutations on mortality *δ* = 0.001 under different mutation rates *M* and different effects of mutations on production rate *λ*. The vertical lines represent mean age at maturity (solid) with standard deviation (dashed). The thick solid line captures the fraction of remaining reproduction, which is proportional to Hamilton's force of selection.

**Figure 5:**
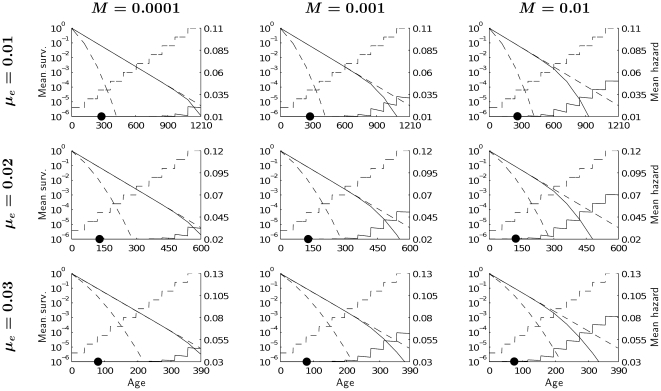
Age-dependence of mean hazard rate (solid lines with steps) and mean survivability (solid decreasing lines) given *δ* = 0.01 and *λ* = 0 for different levels of extrinsic mortality 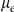
 **and mutation rate** ***M*** **.** Dashed decreasing lines represent survivorship for non-mutated (upper lines) and maximally mutated genotypes (lower lines). The dashed lines with steps represent mortality of maximally mutated genotypes, while mortality of genotypes without mutations lies on the x-axis. Black dots represent mean age at maturity. For all cases the frequency of the maximaly mutated genotype is close to zero or numericaly equal to zero. Note that maximum age 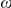 is smaller the higher the level of environmental mortality 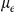.Thus, if 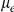 = 0.02, then 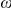 = 600 and if 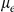 = 0.03, then 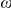 = 390.

Figures 3, 4 and 5 provide evidence that the influence of mutation accumulation on shaping life history patterns can generally be neglected at ages that are relevant for fitness. The frequencies of mutations that accumulate at different ages under different combinations of mutation rates *M* with different magnitudes of effect 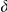 of mutations on mortality (Fig. 3), with different magnitudes of effects *λ* of mutations on reproduction (Fig. 4), and with different levels of background mortality 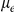, are zero or minor at ages that contribute significantly to evolutionary fitness. Mutations accumulate strongly at ages when selection pressure is low, i.e., when remaining reproduction is less than 1% (Figs. 3 and 4) and when the probability of surviving to that age is low (Fig. 5).

Figure 5 shows that even under conditions that are most favourable for mutation accumulation (small mutational effects on mortality, no mutational effects on reproduction, high mutation rates), less than 1% of a cohort will be alive when mutations start accumulating and raising mortality. For moderate and low mutation rates, the differences between the mean survival curve and the survival curve for non-mutated case is negligible for 99.9% of the population. Although the most mutated genotype exhibits a survival curve that is very distinct from the average, such a genotype is so infrequent that its influence can be neglected.

Note that in Fig. 5, frequencies of accumulated mutations are reflected in the step-sizes of the mortality trajectory. The steps in the pattern of mean mortality are slightly tilted to the right, because the population is heterogeneous with respect to mortality. Since the number of discrete age classes exceeds the number of genes, individuals with high numbers of mutations die earlier within an age-class, leaving the remaining survivors with a lower mean level of mortality. Steps would be strictly horizontal if the number of genes matched the number of discrete age-classes.

Figures 3, 4 and 5 confirm the results shown in Fig. 2: the reduction of the mean age of maturity due to mutation accumulation is minor. The variability of age at maturity across genotypes is very low even for high mutation rates and slightly increases when effects of mutations on mortality or on reproduction become stronger.

Generally, the frequency of mutated genes is low over fitness-relevant ages. It increases with age. This increase is faster the higher the mutation rate (Fig. 3), and it is slower, the larger the adverse effects of mutations on either mortality or reproduction (Fig. 3 and 4). A higher background death rate increases the frequency of mutated genes expressed early, but comparing mortality patterns across rows (i.e., across different magnitudes of background mortality) in Fig. 5 reveals that its role is relatively minor.

**Figure 6:**
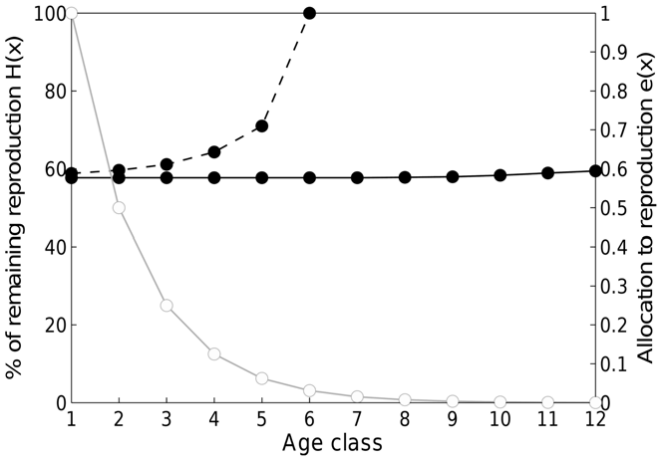
Importance of mutation accumulation vs. optimization in the model by Charlesworth [**21**]. Black circles indicate allocation to reproduction e(x) for a non-mutated phenotype (solid line) and for a phenotype loaded with mutations (dashed line). White circles represent the level of remaining reproduction left to an individual at that age (which captures selection pressure against deleterious mutations). Without mutations the optimal allocation patterns is approximately constant. With mutation accumulation, strong differences in allocation strategy between mutated and non-mutated phenotype appear at ages when the percentage of lifetime reproduction left to an organism has fallen below 5%. (Calculations based on Table 2 by Charlesworth [21]).

Last but not least we took a particular result by Charlesworth [21] a step further, which is necessary to shed light on the main question of our paper. Based on the numbers given in Table 2 of his article, our Figure 6 demonstrates the fitness relevance of ages when mutation accumulation disturbs optimal life history patterns significantly. We find that in Charlesworth's model, mutation accumulation seems to be important in shaping life history traits only at ages that contribute little to fitness. Over the part of life history that is important to fitness, i.e., when the death of an individual of that age would imply a loss of many potential offspring (in his model ages 0 to 4), the value of the optimal life history trait is changed little by the influence of deleterious mutations. Though Charlesworth's model, based on quantitative genetics, is very different from ours, results of both models are consistent: the optimal life history pattern without adverse mutations does not diverge significantly from the optimal life history pattern under a load of mutations.

## Discussion

### Discussion of the Results

Baudisch [24] hypothesized that mutation accumulation may be a minor force in shaping life histories. Our results support this hypothesis. In accordance with the prediction of mutation accumulation theory [1], [4], we find that the frequency of deleterious mutations increases with age. But mutations equilibrate at significant levels only when selection pressure is low, which occurs at ages that contribute negligibly to fitness. Even for the highest mutation pressure (mutation rates of 0.01 per gene or 0.1 per genome), mutation accumulation reduced mean optimal age at maturity and mean fitness by less than 7% compared with the non-mutated genotype.

We found an inverse relation between strength of the effect of a mutation and the equilibrium frequency of accumulated mutations. Selection pressure opposes an increase in mortality equally strongly, either for many small or for few big mutations. In shaping the age at maturity and mutational load, the effect size of mutations played a minor role. The parameter that captures the constant background mortality in our model had virtually no effect on the qualitative profile of mutation accumulation. It strongly affects lifespan and age at maturity, but this is not surprising. Lifespan is inversely related to mean mortality, and if lifespans are short, maturity must occur early to ensure sufficient reproduction. High environmental mortality compresses a life history into a shorter time interval. Phrasing this finding in terms of “pace” and “shape,” two concepts that have recently been suggested by Baudisch [41], background mortality determines the pace of mutation accumulation, but it does not affect the shape of mutation accumulation. The “relative age” (in term of proportion of total lifespan) at which mutations equilibrate at significant frequencies remains the same for different background mortality levels. For different levels of background mortality, the age when all or almost all genes are mutated corresponds consistently to ages when remaining expected at birth reproduction is very low. We found the optimal age at maturity to occur roughly at 25% of maximum lifespan (consistent with the idea of life history invariants proposed by Charnov, e.g., [42], [43] and [34]).

### Discussion of the Model

As new features, representing an organism's genotype by a bit-string, we explicitly include size and its effect on reproduction via energy production within an optimal resource allocation model to study the evolution of senescence under mutation selection balance. In our model, a mixture of many genotypes reaches mutation-selection equilibrium. At this equilibrium most genotypes can still mutate, but the frequencies remain unchanged because of the counterbalance of selection. Genotypes with higher mutational load have lower chances of surviving and thus lower fitness and contribute less to the next generation; hence their frequencies decline. Equilibrium is maintained. Since the model operates on frequencies, population size does not matter.

The model is based on ten age-classes (corresponding to 10 genes), because this number was the best compromise between the speed of computations and precision of the results. To evaluate the influence of the number of genes on outcomes, we ran the analysis for several different numbers of genes, holding the genome-wide mutation rate constant. In models with larger number of genes the age-specific mutation rate *M* was set at a correspondingly lower value. We found that both mutational load and age at maturity were only weakly sensitive to the number of genes. Allowing for smaller age-classes and correspondingly more age-dependent genes led to smoother mortality patterns, but it did not alter their qualitative shape. We are confident that our choice of gene number does not bias our results, since each “gene” can be split into smaller parts with a simultaneous decrease of the age-specific mutation rate. Consequently, our model results should hold assuming many genes with small effects or few genes with large effects.

We assume that mutated genes increase mortality additively. Theoretically it is possible that different combinations of mutations could together generate detrimental effects that could be not only additive. While in the future it might be interesting to test a non-additive model, we do not believe that it would lead to qualitatively different results.

The highest genome-wide mutation rate per generation assumed in our calculations was 0.1. In nature, the mutation rates per total effective genome per sexual generation were estimated for *Mus musculus* as 0.9, for *Homo sapiens* as 1.6, for *Drosophila melanogaster* as 0.14 and for *Caenorhabditis elegans* as 0.036 [44], or by indirect methods: *Drosophila* as 0.3–0.5 and mammals around 1.0 [45]. We argue that the value 0.1 chosen in our model is high, because only a fraction of genes act in an age-specific manner. A value of 0.1 for humans implies that fewer than 6.3% of genome-wide mutations have age-specific effects, while for *Drosophila melanogaster* this value of 0.1 implies that more than 71% of mutations have age-specific effects. The existence of a subset of genes with age-specific effects has been confirmed in large-scale demographic-studies for novel germ-line mutations on mortality rates in *Drosophila melanogaster* and *Caenorhabditis elegans* [18], [19], [46]–[Bibr pone.0034146-Golden1] [48] (see [49] for review) but they do not give an estimate for the percentage of genome wide mutations that are age-specific. Pedro de Magalhaes et al. [50] in their extensive meta-analysis of age-related gene expression profiles for mice, rats and humans (about 5mln gene expression measurements) found only 63 genes that change their expression with age. Given that the number of protein-coding genes of mice, rats and humans are at the magnitude of 20000–25000 genes [51], [52], 63 is small. If the Magalhaes et al.'s results reflect the true percentage of age-specific genes, then the impact of mutation accumulation might be even weaker than suggested by our results.

Previous models of mutation accumulation to explain the shape of mortality patterns are based on the approach by Hamilton [4]. These models consider mutation-selection equilibrium at each age separately, assuming a marginal change in mortality at one age while all other ages remain unaffected. The age-specific mutational pattern is then derived by combining the mutational load found for each age. Such a “linear” approach has been criticized by Wachter and colleagues [10] who developed a more general approach. In our model the mutation profile across all ages affects the selection pressure for each single age, thus our model is in this sense nonlinear, because it allows for mutations to accumulate simultaneously at all ages. We believe that allowing a multitude of genotypes with their specific mortality patterns and fitness values competing with each other makes our model more realistic than previous models.

In our model we assume population size to be constant but there is no generation overlap, thus no specific assumptions about density dependence are necessary [53] (Please compare with Charlesworth's model [21] where overlapping generations were assumed and population size was controlled just after birth). The assumption about non-overlapping generations is introduced for simplicity. It allows us to use classic life-history optimization methods where the net reproductive rate is maximized. Including overlapping generations in our model would be difficult and would complicate the understanding of the model. We expect that including overlapping generations would mainly prolong the time (number of generations) that is needed to achieve mutational-selection equilibrium but would not much alter the equilibrium itself. We thus do not believe that our results would be affected significantly.

The model presented here assumes that the optimal age at maturity varies depending on the genotype, i.e., for every genotype a corresponding optimal age at maturity is calculated given levels of background and internal mortality. In this way, we allow age at maturity to be influenced by environmental conditions and by epistasis. Empirical evidence for age-specific epistatic effects (age-specific change in *gene*×*genetic background* interactions) is given in a study of *Drosophila melanogaster* by Spencer and Promislow [49]. We assume that every individual with a specific genotype matures at the same age. Allowing for variance in the age at maturity within the same genotype would complicate our model greatly. Given, however, that the variance in optimal age at maturity across genotypes is close to zero for most of the cases studied (Fig. 3), we expect that assuming zero variance in the optimal strategy within a genotype does not restrict our results significantly. Having virtually the same optimal age of maturity for all genotypes, mutational variance of this trait inside one genotype should not play an important role, especially when we expect strong stabilizing selection [21]. In this case, the assumption of an epistatic interaction between age at maturity and mutational background (genotype) may be not necessary.

Another innovative feature in our scenario of our model is that mutations have dual negative effects on energy acquisition and allocation. These effects act indirectly via mortality or via production rate. Mutations directly increase mortality and (eventually, if assumed) decreases production rate. Given lower survival chances, the optimal age at which energy allocation switches from growth to reproduction (maturity) happens earlier; early maturity means less time for growth and thus a lower size at maturity; smaller size means lower energy acquisition. We can observe the same situation for lowered production rate. Concluding, both allocation and acquisition can be affected by mutations. The effect of mortality on optimal life history patterns can be found in many papers e.g., [42], [54] and [30], please see also section “discussion of the results”.

### Conclusions

We conclude that mortality patterns over fitness relevant ages are mainly determined by life history tradeoffs. Mutation accumulation could have a significant impact on senescence patterns only if mutations would significantly alter those tradeoffs. But this is unlikely, as Charlesworth [21] found that genetic correlations (and thus tradeoffs) seem to be largely unaffected by mutation accumulation except under extreme conditions. Our model provides evidence that mutation accumulation may be responsible for a rapid increase of mortality at the end of life. This increase appears, however, at ages that are unimportant for fitness. Humans today survive to ages at which reproduction is impossible or rare, though indirect contributions to fitness resulting from care of offspring and the offspring of offspring may still be significant. Hence, the age trajectory of mortality for modern humans may be shaped in part by mutation accumulation at old and oldest-old ages. But for other species and for humans over most of their existence, our results suggest that the role of mutation accumulation in shaping adult (but not senile) mortality patterns and earlier life history traits, such as age at maturity, is negligible. Background mortality (not dependent directly on mutation accumulation and indirectly on age) is mostly responsible for both age at maturity and the pace of deleterious mutation accumulation, whereas it is almost neutral for the shape of the accumulation.
